# Prone Positioning Is a Feasible Approach in the Diagnostic Work-Up of Posterior Pulmonary Nodules and a Means to Limit CT-to-Body Divergence: A Retrospective Cohort Study

**DOI:** 10.3390/diseases14060198

**Published:** 2026-06-02

**Authors:** Russell Vo, Tristan Post, Daniel Smith, Valerie Peters, Isha Puri, J. W. Hollingsworth, Sai Karan Vamsi Guda

**Affiliations:** 1 Department of Internal Medicine, Texas Health Harris Methodist Hospital, Fort Worth, TX 76104, USA; 2 U.S. Anesthesia Partners, Fort Worth, TX 76104, USA; 3 Department of Internal Medicine, Texas Christian University Burnett School of Medicine, Fort Worth, TX 76104, USA

**Keywords:** CT-to-body divergence, ion robotic bronchoscopy, posterior lung nodules, anesthesia-induced atelectasis, prone positioning, lung cancer

## Abstract

**Background:** Lung cancer is the second most common cause of cancer with high mortality, thereby emphasizing the importance of early detection. However, the rate of new lung cancer diagnosis has remained relatively unchanged. Despite the advancements in navigational bronchoscopy, the diagnostic yield of pulmonary nodules, particularly posterior nodules, is often limited by CT-to-body divergence. Our study aims to evaluate the feasibility and safety of prone positioning during navigational bronchoscopy and its impact on the diagnostic yield of posterior pulmonary nodules. **Methods:** Retrospective cohort study of nine patients who underwent Ion robotic navigational bronchoscopy in prone position and 237 patients in supine position. The study period was July 2024 to December 2024 for the prone cohort and July 2020 to September 2024 for the supine cohort. **Results:** In the supine cohort, the diagnostic yield was 93.3%, including a malignant yield of 62.3%, and the post-operative complication rates were 1.5% for pneumothorax, 3.5% for bronchopulmonary hemorrhage, and 1.9% for respiratory failure. In the prone cohort, the diagnostic yield was 77.8% and a malignant yield of 85.7%, and the postoperative complication rates were 0% for pneumothorax, bronchopulmonary hemorrhage, and respiratory failure. **Conclusions:** CT-to-body divergence is a major obstacle in the diagnostic work-up of pulmonary nodules, of which a major contributor is atelectasis. Our study demonstrates that prone positioning combined with a strict anesthesia protocol is both a feasible and safe approach in the diagnostic work-up of pulmonary nodules.

## Simple Summary

Lung cancer remains the leading cause of cancer-related mortality globally, and the diagnostic yield for pulmonary nodules, especially posterior lesions, continues to be limited by CT-to-body divergence despite advances in navigational bronchoscopy. In this retrospective study, we report data on supine and prone cohorts and aim to determine the feasibility of prone positioning in the diagnostic work-up of posterior pulmonary nodules in patients undergoing Ion robotic bronchoscopy.

## 1. Introduction

Lung cancer is the second most common cancer and is the leading cause of cancer-related deaths in both the United States and worldwide [1]. The overall 5-year survival rate is 22%, but with early detection, survival rates can be as high as 90%, thereby underscoring the importance of early detection [2,3]. The widespread adoption of computed tomography (CT) scans for lung cancer screening has increased the detection rate of pulmonary nodules from 3.9 to 6.6 per 1000 person-years; however, the rate of new lung cancer diagnosis has remained relatively unchanged [1,4]. While most pulmonary nodules are benign, those that are suspicious of malignancy require careful evaluation with the least invasive method recommended [4]. Depending on the size and location of the pulmonary nodule, this process can be challenging, and a significant hurdle with any technique of bronchoscopy is the ability to achieve a high diagnostic yield of the lesion in question [5]. Despite the advancements in bronchoscopy, including navigational bronchoscopy, the wide-ranged diagnostic yield of pulmonary nodules has been historically limited by CT-to-body divergence [6,7].

Navigational bronchoscopy provides a minimally invasive and relatively safe means (as compared to traditional transbronchial needle biopsy) to access hard-to-reach pulmonary lesions [8]. However, all current guided bronchoscopy systems rely on a pre-procedural CT scan to create a virtual map of the patient’s airways. Any change in lung anatomy between the pre-procedural static CT scan and the bronchoscopy procedure can lead to divergence between the expected and actual location of a pulmonary nodule in a dynamic, breathing lung [6,9]. This divergence is referred to as “CT-to-body divergence,” which is a major obstacle in the field of bronchoscopy, and can limit the diagnostic yield, prolong procedure time, and be technically challenging to the operator. There are several factors contributing to CT-to-body divergence, including lung volume variation (e.g., full inspiration) during pre-procedural imaging, anatomic changes due to patient positioning, presence and development of atelectasis intra-procedurally, nodule motion, ventilator protocol, and ferromagnetic objects (e.g., anesthesia equipment, intravenous poles, fluoroscopy units, etc.) [6,10,11].

Anesthesia-induced atelectasis is a major contributor to CT-to-body divergence, and occurs in most, if not all, patients within minutes of induction. The dependent regions of the lungs and particularly posterior pulmonary nodules are most disproportionately and most prominently affected [12,13]. In fact, Sagar et al. reported an impressive 89% of patients undergoing bronchoscopy had atelectasis, with more than 50% occurring in the dependent areas of the lung [12]. Similarly, Casal et al. reported atelectasis in dependent areas of the lung in 40% of patients undergoing cone beam CT-guided bronchoscopy, of which atelectasis completely obscured nodule visualization in 20% of cases [13]. The mechanism of anesthesia-induced atelectasis is multi-fold. This includes compression atelectasis as observed by diaphragmatic relaxation secondary to anesthetic, absorption atelectasis in the setting of alveolar displacement of nitrogen, and adhesive atelectasis due to impairment of pulmonary surfactant [14,15]. The increased presence of atelectasis results in regional loss of lung aeration, airway narrowing, and distortion of peripheral bronchial anatomy, thereby reducing the accuracy of virtual navigation platforms that are dependent on preserved airway architecture [16].

Patient positioning plays an important role in the development of atelectasis and the extent of CT-to-body divergence. Specifically, positioning influences regional lung mechanics, diaphragmatic excursion, chest wall compliance, pulmonary blood flow distribution, and dependent alveolar ventilation [17]. These physiologic changes become increasingly important during general anesthesia, when loss of spontaneous respiratory effort and neuromuscular blockade predispose patients to alveolar collapse [18,19]. In the supine position, the dependent, posterior lung regions are particularly susceptible to atelectasis due to shifting of abdominal contents, reduction in thoracic volume, and pooling of blood [20,21]. Furthermore, supine positioning has been shown to decrease functional residual capacity (FRC) by 44% as compared to a 12% reduction in the prone position [14]. Consequently, supine positioning may contribute to reduced lesion visibility and greater CT-to-body divergence, especially in posterior pulmonary nodules. In contrast, prone positioning is a well-known and established strategy to reduce the degree of atelectasis by improving the FRC and ventilation and decreasing lung compression as seen in the established approach to the management of acute respiratory distress syndrome (ARDS) [22,23,24].

The physiologic advantages seen in prone positioning may provide a means to improve procedural accuracy and diagnostic performance, particularly in the evaluation of posterior pulmonary nodules where dependent atelectasis and CT-to-body divergence may negatively impact lesion localization. By potentially reducing posterior lung compression and improving aeration in dependent lung regions, prone positioning may enhance lesion visualization and navigation accuracy during navigational bronchoscopy. However, these potential benefits must be carefully balanced against the practical and procedural challenges associated with prone positioning. It is important to consider airway management as prone positioning may complicate airway access and security, particularly in patients with difficult airway anatomy, elevated body mass index, and may not be ideal in patients at risk for emergent airways or difficult re-intubation [23,24]. In addition, physiologic changes associated with prone positioning must be considered, which includes alterations to chest wall compliance, venous return, airway pressure, and pulmonary mechanics, thereby affecting both hemodynamic stability and ventilatory management during prolonged procedures. Another consideration and potential challenge with prone positioning includes the development of pressure injuries. Additionally, prone positioning may introduce procedural and logistical challenges including technically challenging procedures, prolonged procedure time, and further risk of procedure-related complications), as well as management of procedure-related complications in the prone position [25]. Furthermore, as a novel procedural approach, it may impact workflow efficiency and resource allocation within the bronchoscopy suite and periprocedural care teams.

In this single-center, retrospective cohort study, we identified patients undergoing robotic navigational bronchoscopy for diagnostic work-up of posterior pulmonary nodules in both the supine and prone position. We aimed to determine the feasibility of prone positioning during navigational bronchoscopy with the use of a strict anesthesia protocol and its impact on the diagnostic yield of posterior pulmonary nodules.

## 2. Materials and Methods

### 2.1. Study Design

We conducted a retrospective cohort study including electronic medical record review of 237 patients who underwent robotic navigational bronchoscopy in the supine position and nine patients in the prone position in the diagnostic work-up of posterior pulmonary nodules. The study period for the supine cohort was from July 2020 to September 2024. The study period for the prone cohort was from July 2024 to December 2024. The decision to limit enrollment of the prone cohort was intentional, allowing for a systematic evaluation of procedural logistics, airway safety, anesthetic reproducibility, and operator ergonomics prior to broader implementation. Given the novelty of prone robotic bronchoscopy, a stepwise adoption strategy was favored to identify unforeseen technical or safety concerns before expanding patient enrollment. A posterior pulmonary nodule was defined as a subpleural nodule, as defined on CT imaging, of less than 30 mm in diameter. All cases utilized the Ion^TM^ robotic bronchoscopy system (Intuitive Surgical, Sunnyvale, CA, USA). All cases took place at the Texas Health Harris Methodist Hospital in Fort Worth, TX. Patients were individually evaluated for suitability for prone positioning. Inclusion criteria for prone positioning were patients > 18 years old with a posterior pulmonary nodule who consented to prone positioning. Patients with contraindications to prone positioning were generally not considered candidates and thus excluded. Absolute contraindications included unstable spinal pathology or fractures, while relative contraindications included recent trauma or surgery, elevated intracranial pressure, hemodynamic instability, anticipated ventilation difficulties, difficult airway anatomy, or severe obesity (without a specific cutoff). We collected data including demographics, comorbidities, nodule size, characteristics, location, procedure details, pathology, postoperative complications, and interval imaging and procedures. Diagnostic yield (DY) was calculated as the proportion of all patients undergoing a procedure in whom a specific malignant or definitively benign diagnosis is established, in accordance with the American Thoracic Society (ATS) [26,27]. Malignant yield (MY) was calculated as the proportion of malignant diagnoses out of the total proportion of patients with a definitive diagnosis. This study was approved by the University of Texas Southwestern institutional review board #STU20251500.

### 2.2. Anesthesia Considerations

In the pre-procedural phase, all patients received incentive spirometry, treatment with a direct-acting bronchodilator (e.g., albuterol), and treatment with a muscarinic antagonist (e.g., glycopyrrolate) to bolster alveolar recruitment and minimize atelectasis. The use of benzodiazepines and narcotics was avoided throughout the procedure to minimize the risk of respiratory depression in the post-procedural period. In patients requiring anxiolysis, a selective alpha-2 agonist (e.g., dexmedetomidine) was preferred for its minimal effect of respiratory depression and added benefit of reducing sympathetic stimulation from intubation and bronchoscopy [5]. In the induction phase, all patients underwent general anesthesia, and were administered 1–2 mg/kg propofol, 0.1 mg/kg vecuronium, 1–1.5 mg/kg lidocaine, and titrated esmolol for a narcotic-free induction. Intubation was performed expeditiously using video laryngoscopy while patients were in the supine position. The endotracheal tube (ETT) size was 8.5 mm internal diameter for females and 9.0 mm for males. The use of a larger bore ETT allowed for easier passage of the bronchoscope and delivery of larger tidal volumes (TV) while avoiding excess airway pressure [5]. Current guidelines recommend <80% fraction of inspired oxygen (FiO_2_) for robotic bronchoscopy to reduce the degree of reabsorption atelectasis [28]. Continuous positive airway pressure (CPAP) was used both prior to and throughout induction, and patients were routinely able to be induced with 21% FiO_2_. In the maintenance phase (after induction), patients were ventilated with 7–12 cmH_2_O of positive end expiratory pressure (PEEP) and TV 10–12 mL/kg of ideal body weight. Recruitment maneuvers were administered to facilitate reversal of any degree of atelectasis developed during induction. While not necessarily contraindicated, volatile anesthetics were avoided to reduce excessive exposure of operating room staff in the setting of repeated access of the circuit for introduction of the scope throughout the procedure. Total intravenous anesthesia (TIVA) and further paralysis were therefore employed using propofol and vecuronium, respectively. Procedural imaging was done with the lungs at full tidal inspiration, which was achieved using an adjustable pressure-limiting (APL) valve. The duration of breath hold ranged from 5 to 10 s to allow for complete lung inflation and minimalization of motion artifact [14]. The use of an experienced multidisciplinary team, rather than ad hoc personnel, promoted familiarity with the standardized anesthesia protocol described above and minimized delays during induction, positioning, and bronchoscope registration, thereby helping preserve lung recruitment and limit the extent of intraoperative derecruitment. In particular, experienced anesthesia providers ensured consistent adherence to the protocol and optimization of peri-procural ventilatory management.

### 2.3. Prone Positioning

**Figure 1:**
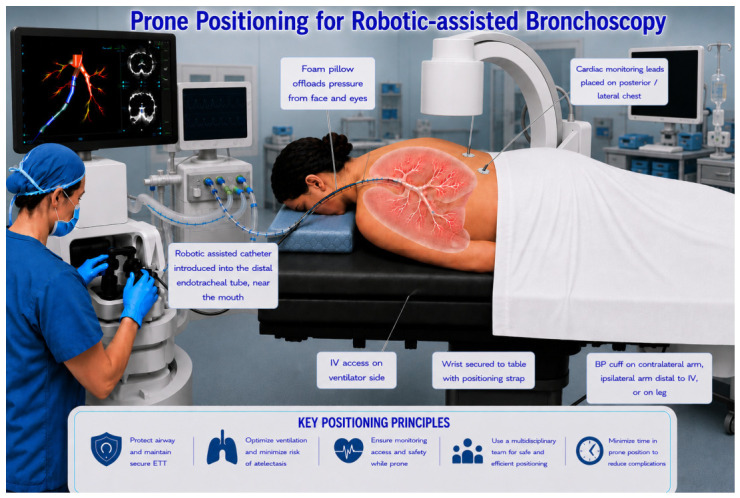
Patient positioning, procedural set up, and monitoring during prone robotic-assisted bronchoscopy.

Once intubated, surgical or twill tape was used to secure the airway, depending on provider preference. The airway was secured towards the side of the patient that faced the ventilator while in the prone position. Then, the patient was positioned prone with a multidisciplinary team (Figure 1). Procedure staff used a commercially available foam pillow designed for the use of prone positioning to offload pressure from the patient’s eyes and face to avoid excessive pressure on the airway. If not contraindicated, intravenous (IV) access was placed on the side of the patient that faced the ventilator while in the prone position. Cardiac monitoring leads were placed on the posterior and lateral aspects of the patient while prone. A non-invasive blood pressure cuff was placed on either the contralateral arm to the IV catheter, on the ipsilateral arm distal to the IV catheter, or on the leg to avoid interruption of the TIVA infusion when inflated. Careful planning of ergonomics allowed for rapid positioning of the patient, thereby minimizing the time in which atelectasis could develop.

### 2.4. Procedure

On the day of the procedure, imaging via CT scan of the chest without contrast was performed for procedural planning and reviewed immediately prior to the procedure. Once the patient was intubated and in the prone position, a fiberoptic bronchoscopy was introduced with direct visualization through the mouth via the ETT and then advanced to the tracheobronchial tree, where a full tracheobronchial tree survey was performed. Registration was done with the Ion robotic navigational system. Afterwards, the operator performed bronchoscopic inspection of the distal trachea and carina, followed by first the right, and then the left main bronchus, lobal segmental to subsegmental level bronchi for endobronchial masses, lesions, and foreign bodies. Ion robotic navigational bronchoscopy was used to localize the posterior lesion. 3D fluoroscopy was used to confirm the catheter’s proximity to the lesion. Radial endobronchial ultrasound was used to confirm adequate location of the catheter tip prior to biopsies performed. Under fluoroscopic guidance, biopsies were performed using fine needle aspiration (FNA) and/or transbronchial biopsy (TBBx) using forceps, a cryoprobe, or a bronchial brush. The samples were collected for pathology. A bedside pathologist was present for rapid on-site evaluation (ROSE) to confirm adequate tissue sampling as well as preliminary diagnosis. If indicated, linear EBUS was then performed to survey lymph nodes, and if necessary, biopsies were performed. If indicated, bronchoalveolar lavage (BAL) was then performed. All post-biopsy bloody secretions were aspirated dry. The tracheobronchial tree was surveyed for active bleeding both during and at the culmination of the procedure. The bronchoscope was subsequently removed. Fluoroscopy was used to confirm the absence of pneumothorax at the end of the procedure.

## 3. Results

### 3.1. Demographics

In the supine cohort, there were a total of 252 Ion robotic bronchoscopies performed across 237 patients, of which 14 patients underwent repeat procedures (Table 1). In the prone cohort, there were a total of nine patients who underwent a singular Ion robotic bronchoscopy. The supine cohort was composed of 114 (45.3%) males and 142 (55.4%) females with the mean age of 71.8 ± 10.5 years and a mean body mass index (BMI) of 27.1 ± 6.1 kg/m^2^. The prone cohort was composed of five (55.6%) males and four (44.4%) females with the mean age of 69.6 ± 5.9 years and BMI 30.2 ± 7.6 kg/m^2^. In the supine cohort, the most prevalent comorbidities included hypertension (74.2%), tobacco use (64.3%), family history of malignancy (60.5%), chronic obstructive pulmonary disease (COPD) (49.6%), personal history of malignancy (48.4%), and diabetes mellitus (23.4%). In the prone cohort, the most prevalent comorbidities included hypertension (88.9%), tobacco use (77.8%), family history of malignancy (66.7%), diabetes mellitus (55.6%), personal history of malignancy (55.6%), and COPD (33.3%).

### 3.2. Procedure Results

In the supine cohort, there was a total of 256 pulmonary nodules biopsied, of which 153 (59.7%) were in the right lower lobe (RLL) and 103 (40.3%) in the left lower lobe (LLL), with a mean nodule size of 21.1 ± 11.6 mm (Table 2). Data regarding nodule radiodensity, Hounsfield units (HU), spiculation, and the presence of the bronchus sign was not collected for the supine cohort. In the prone cohort, all patients underwent a singular pulmonary nodule biopsy, of which six (66.7%) were in the RLL and three (33.3%) were in the LLL. The mean nodule size was 18.3 ± 9.3 mm and radiodensity was 56.2 ± 32.6 HU. On preoperative CT imaging, it was noted that one (11.1%) of the pulmonary nodules was spiculated, and a bronchus sign was present in one (11.1%) of the pulmonary nodules. For the supine cohort, the mean total procedure duration was 48.0 ± 19.0 min, of which the mean fluoroscopy time was 3.4 ± 2.7 min. For the prone cohort, the mean total procedure duration was 60.8 ± 14.2 min, of which the mean fluoroscopy time was 4.4 ± 2.1 min. In the supine cohort, 70.3% of patients underwent multiple biopsy modalities, which included fine needle biopsy (FNA) in 88.9% of cases and TBBx in 77.0% of cases. The mean number of biopsy attempts (passes) in the supine cohort was 6.4 ± 3.9 passes. Furthermore, radial EBUS, linear EBUS, BAL, and fiduciary placement were performed in 93.2%, 55.6%, 92.6%, and 7.2%, respectively. Rapid on-site evaluation by a pathologist was available and used in 96.2% of cases in the supine cohort. This is compared to the prone cohort, in which 88.9% of patients underwent multiple biopsy modalities, which included FNA in 88.9% of cases and TBBx in 100% of cases. The mean number of biopsy attempts in the prone cohort was 6.7 ± 3.1 passes. Radial EBS, linear EBUS, BAL, and fiduciary placement were performed in 100%, 66.7%, 77.8%, and 11.1% of cases, respectively. Rapid on-site evaluation by a pathologist was available and used in 100% of cases in the prone cohort.

### 3.3. Diagnostic Yield and Post-Operative Complications

In terms of definitive pathology findings, the diagnostic yield was 93.3% in the supine cohort with a malignant yield of 62.3% (Table 3). In the prone cohort, the diagnostic yield was 77.8%, of which the malignant yield was 85.7%. The post-operative complication rates for the supine cohort included the following: pneumothorax (1.5%), bronchopulmonary hemorrhage (3.5%), and respiratory failure (1.9%). Furthermore, the incidence of interval imaging studies for surveillance and procedures (e.g., repeat biopsy or surgical intervention) at 1 year was 18.7% and 14.0%, respectively. The post-operative complication rates for prone patients were 0% for pneumothorax, bronchopulmonary hemorrhage, and respiratory failure. The rate of interval imaging post-procedure was 0% and interval procedures (e.g., repeat biopsy or surgical resection) were 11.1%. Supplemental Figure S1 represents the incidence of cancers in both the supine and prone cohorts. Supplemental Figure S2A and S2B represent a sample of the CT scan (axial view) of a posterior pulmonary nodule and intraprocedural fluoroscopic imaging of robotic assisted bronchoscopy, respectively. 

## 4. Discussion

Historically, the diagnostic work-up for pulmonary nodules has been limited by CT-to-body divergence, with anesthesia-induced atelectasis, particularly in the posterior lung regions, being a significant contributing factor. This divergence can impair localization of the lesion, prolong procedure time, decrease the overall diagnostic yield, and increase the incidence of procedure-related complications. A meta-analysis by Pyarali et al. demonstrated a pooled diagnostic yield of 85.2% for robotic-assisted navigation bronchoscopy, and a pooled complication rate of 1.18% for pneumothorax and 0.04% for bronchopulmonary hemorrhage [29,30]. However, pertaining to posterior pulmonary nodules, the diagnostic yield is often lower and can range drastically from 50 to 80% [2,31].

Our retrospective cohort study demonstrated a higher observed diagnostic yield in the supine cohort as compared to the prone cohort. However, the diagnostic yield observed in the prone cohort remained within the range reported in larger studies evaluating navigational bronchoscopy for posterior pulmonary nodules [2,31]. There are several factors that may explain the observed discrepancy in the diagnostic yield between the two cohorts. First, although intentional, the prone cohort had a relatively small sample size, which could affect the reliability and overall generalizability of the observed diagnostic yield. Additionally, unlike conventional supine bronchoscopy, prone positioning alters operator orientation, fluoroscopic perspectives, airway manipulation, and catheter stability, all of which are factors that may influence navigation efficiency and biopsy acquisition. These procedural differences likely contributed to an early learning curve that affected diagnostic performance during initial implementation. Furthermore, in small cohorts, each diagnostic or non-diagnostic procedure has a proportionally greater impact on the overall observed diagnostic yield, and therefore the measured performance may not fully reflect the true procedural capability of the technique.

Despite the lower diagnostic yield, prone positioning was associated with an acceptable safety profile and a lower complication rate as compared to the conventional supine approach. In fact, there were no cases of pneumothorax, significant bronchopulmonary hemorrhage, or respiratory failure in the prone cohort, despite the prolonged procedure times and increased technical complexity. The observed safety profile may be attributable to several factors, including implementation of a strict, standardized anesthesia protocol consisting of lung recruitment maneuvers, targeted ventilation strategies, and limitation of ad hoc anesthesia providers, as well as increased provider awareness and procedural caution during adoption of a novel protocol. Similar to the observations regarding diagnostic yield, these findings should be cautiously interpreted with consideration of the small sample size of the prone cohort and its inherent limitations. Additionally, from a systems perspective, implementation of prone bronchoscopy may require additional staffing resources, procedural planning, and workflow modifications during early adoption phases. Increased procedure duration may affect bronchoscopy suite throughput, anesthesia staffing allocation, and peri-procedural resource utilization. However, these considerations may improve with increasing operator familiarity and protocol standardization over time.

Overall, our findings support that prone positioning is a feasible approach in the diagnostic work-up of posterior pulmonary nodules as a means to limit the extent of CT-to-body divergence. It is important to highlight the need for strict anesthesia protocol given the challenges with prone positioning. Additionally, our study had loose inclusion and exclusion criteria in which patients were evaluated on a case-by-case basis. It is important to consider both the absolute (e.g., unstable spine or fractures) and relative contraindications (e.g., recent trauma or surgery, elevated intracranial pressure, hemodynamic instability, difficulties with ventilation, difficult airway, obesity (due to the need for rapid supination in the event of emergencies), etc.) to prone positioning [23,32].

There are several limitations of our study that warrant consideration. First, the retrospective observational design limits our findings to associations and observations alone. There is limited ability to control confounding variables, including operator experience, lesion complexity, procedural sequencing, and evolving workflow optimization over time. In addition, the study was subject to selection bias, as the prone positioning was selectively implemented in a small exploratory cohort rather than through randomized allocation. Another important limitation was the lack of objective physiologic and imaging data. The study did not systematically collect ventilator parameters such as airway pressures, lung compliance, TV, and PEEP, which may help estimate the degree of airway collapse and intraprocedural atelectasis. Similarly, quantitative assessment of CT-to-body divergence, standardized measurements of atelectatic burden on 3D imaging, and reproducible lesion landmarks such as distance to lung pleura were not available. Comparative imaging analysis between the supine and prone cohort was also not systematically performed, thereby limiting evaluation of differences between the two positioning strategies. Additionally, the small sample size of the prone cohorts substantially limited the study’s overall statistical power, generalizability, and meaningful comparison with the larger supine cohort. The imbalance in the cohort size further restricts interpretation of observed differences between the two groups. Future research should focus on conducting a prospective randomized control trial with standardized ventilatory protocols, systemic imaging assessment, and collection of objective physiologic data to further validate these findings, better characterize the relationship between patient positioning (supine vs. prone) and degree of atelectasis, and optimize procedural strategies for the diagnostic work-up of pulmonary nodules, especially posterior lesions.

## 5. Conclusions

Our retrospective cohort study demonstrates that prone positioning combined with a strict anesthesia protocol is a feasible approach in the diagnostic work-up of posterior pulmonary nodules as compared to the conventional supine positioning. This approach seeks to address one of the major physiologic limitations of advanced bronchoscopy, namely anesthesia-induced atelectasis and subsequent CT-to-body divergence within dependent regions of the lung. Although the observed diagnostic yield in the prone cohort was lower than that of the supine cohort, outcomes remained with ranges previously reported in the literature of posterior pulmonary nodules and were achieved with a comparable safety profile. Importantly, our findings highlight the potential role of patient positioning and anesthetic optimization as modifiable procedural variables capable of influencing bronchoscopic accuracy and procedural outcomes. Continued refinement of workflow ergonomics, procedural standardization, and operator experience may further improve the performance of navigational bronchoscopy in the prone position.

This approach aims to limit the extent of CT-to-body divergence, of which a major contributor is anesthesia-induced atelectasis. Prospective studies are warranted to validate these preliminary findings and define optimal procedural and anesthetic protocols for maximizing diagnostic accuracy and limiting procedural complications in the work-up of posterior pulmonary nodules.

## Figures and Tables

**Table 1:** Patient demographics and comorbid conditions in the supine and prone cohorts.

	Supine *n = 252*	Prone *n = 9*
**Demographics**		
Age (mean) (years)	71.8 ± 10.5	69.6 ± 5.9
Male (%)	45.3%	55.6%
BMI (mean) (kg/m^2^)	27.1 ± 6.1	30.2 ± 7.6
**Comorbidities**		
Hypertension (%)	74.2%	88.9%
Diabetes mellitus (%)	23.4%	55.6%
COPD (%)	49.6%	33.3%
Tobacco use (%)	64.3%	77.8%
Hx of malignancy (%)	48.4%	55.6%
FHx of malignancy (%)	60.5%	66.7%

* There were 237 patients in the supine cohort, of which 14 patients underwent repeat procedure.

**Table 2:** Posterior pulmonary nodule lesion characteristics and procedural details in the supine and prone cohort.

	Supine *n = 256*	Prone *n = 9*
**Nodule Characteristics** *		
Nodule size (mean) (mm)	21.1 ± 11.6	18.3 ± 9.3
Location		
RLL (%)	153 (59.7%)	6 (66.7%)
LLL (%)	103 (40.3%)	3 (33.3%)
**Procedure Details** *		
Total Duration (mean) (min)	48.0 ± 19.0	60.8 ± 14.2
Fluoroscopy Duration (mean) (min)	3.4 ± 2.7	4.4 ± 2.1
Biopsy Modality		
FNA (%)	88.1%	88.9%
TBBx (%)	77.0%	100%
# of Biopsy Attempts (passes) (mean)	6.4 ± 3.9	6.7 ± 3.1
Radial EBUS (%)	93.2%	100%

* There were 252 patients in the supine cohort, but a total of 256 nodules were biopsied.

**Table 3:** Pathology results, procedural complications, and longitudinal follow-up in the supine and prone cohort.

	Supine *n = 256*	Prone *n = 9*
**Pathology**		
Benign	90 (35.1%)	1 (11.1%)
Malignant	149 (58.2%)	6 (66.7%)
Inconclusive	17 (6.6%)	2 (22.2%)
**Post-procedure Complications**		
Pneumothorax	4 (1.5%)	0 (0%)
Bronchopulmonary hemorrhage	9 (3.5%)	0 (0%)
Respiratory failure	5 (1.9%)	0 (0%)
**Longitudinal Follow-up**		
Interval surveillance imaging	48 (18.7%)	0 (0%)
Interval procedures	36 (14%)	1 (11.1%)

## Data Availability

The raw data supporting the conclusions of this article will be made available by the authors on request.

## Supplementary Materials

The following supporting information can be downloaded at: https://www.mdpi.com/article/10.3390/diseases14060198/s1, Figure S1: Distribution of cancer diagnosis as seen on pathology; Figure S2: 2A. Axial slice of CT chest without contrast in the lung window demonstrating a 6.7 mm peripheral pulmonary nodule of a patient in the prone cohort; 2B. Intra-procedural fluoroscopic image of robotic-assisted bronchoscopy catheter at the site of peripheral pulmonary nodule of a patient in the prone cohort.

## Abbreviations

The following abbreviations are used in this manuscript:

APL	Adjustable pressure-limiting
ARDS	Acute respiratory distress syndrome
ATS	American thoracic society
BAL	Bronchoalveolar lavage
CPAP	Continuous positive airway pressure
CT	Computed tomography
DY	Diagnostic yield
EBUS	Endobronchial ultrasound
ET	Endotracheal
FiO2	Fraction of inspired oxygen
FNA	Fine needle aspiration
FRC	Functional residual capacity
IV	Intravenous
LLL	Left lower lobe
MY	Malignant yield
PEEP	Positive end expiratory pressure
RLL	Right lower lobe
ROSE	Rapid on-site evaluation
TBBx	Transbronchial biopsy
TIVA	Total intravenous anesthesia
TV	Tidal volume
