# Supplementary material for: Prone Positioning Is a Feasible Approach in the Diagnostic Work-Up of Posterior Pulmonary Nodules and a Means to Limit CT-to-Body Divergence: A Retrospective Cohort Study
Source: Diseases. 2026 Jun 2;14(6):198. doi: 10.3390/diseases14060198 (PMC13298979; doi:10.3390/diseases14060198)
Supplement: Supplementary file 1 [file diseases-14-00198-s001.zip › diseases-4295000-supplementary.pdf]

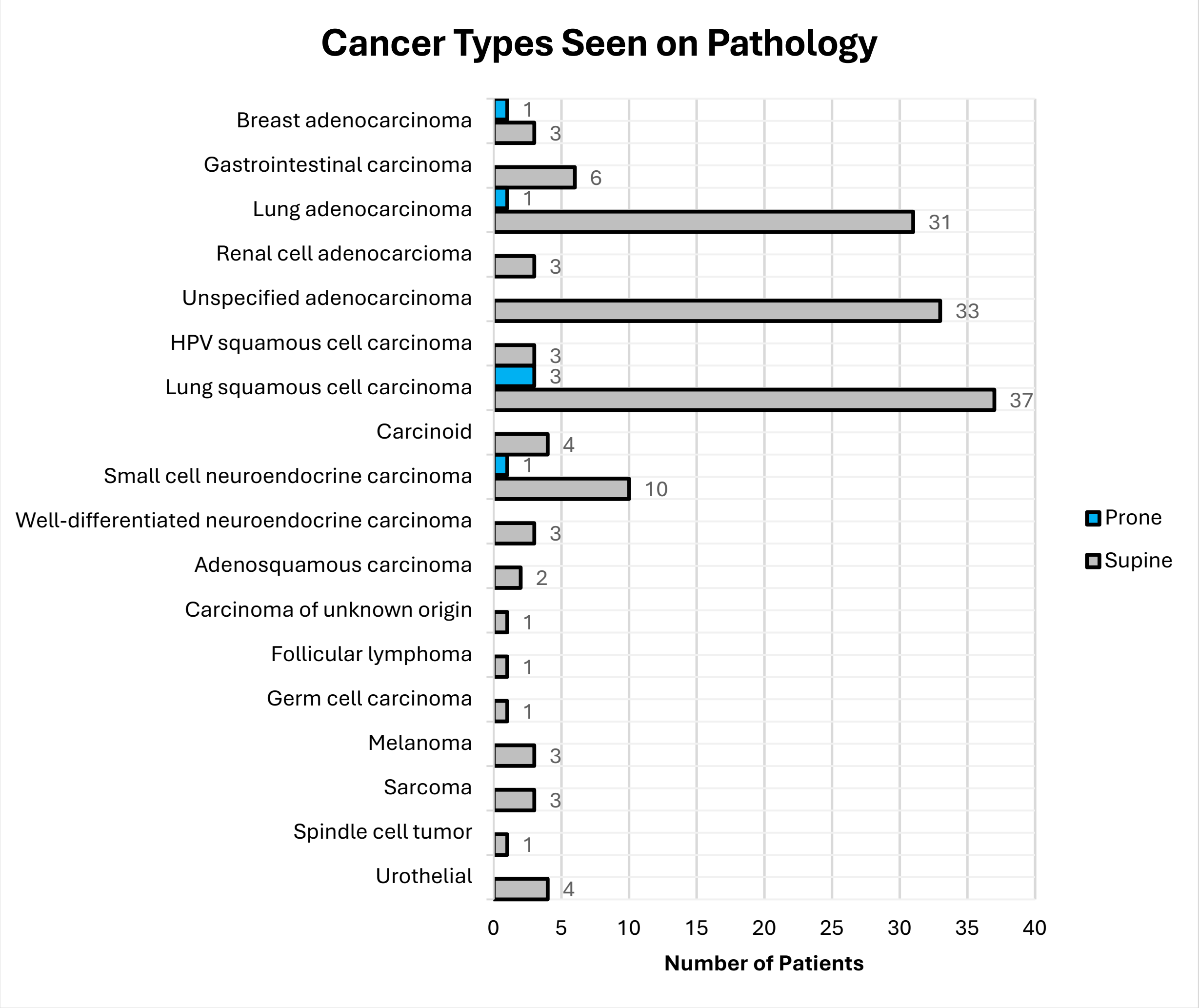

Supplemental Figure S1. Distribution of cancer diagnosis as seen on pathology.

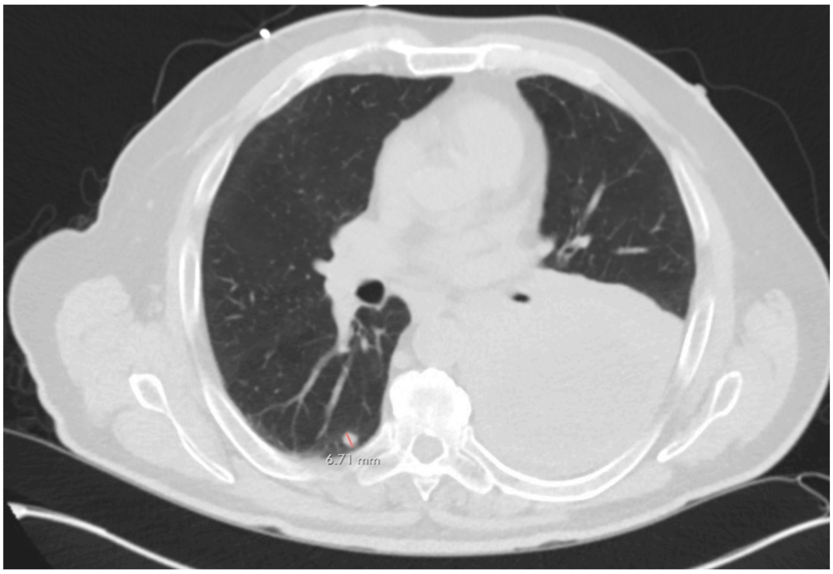

Supplemental Figure S2A. Axial slice of CT chest without contrast in the lung window demonstrating a 6.7 mm peripheral pulmonary nodule of a patient in the prone cohort.

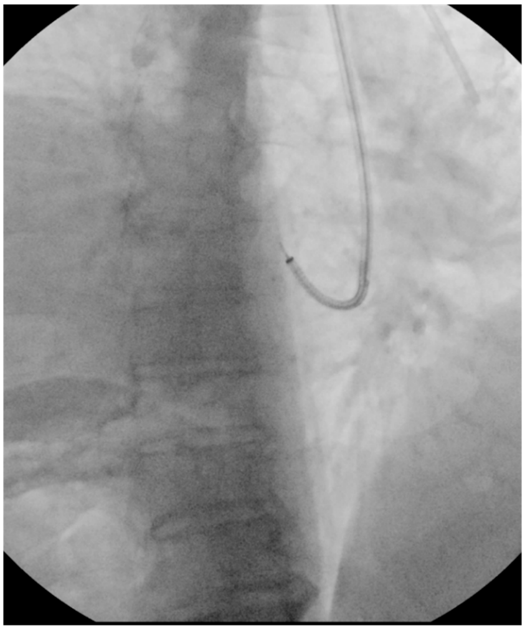

Supplemental Figure S2B. Intra-procedural fluoroscopic image of robotic-assisted bronchoscopy catheter at the site of peripheral pulmonary nodule of a patient in the prone cohort.
